# Enhanced (*t*, *n*) threshold *d*-level quantum secret sharing

**DOI:** 10.1038/s41598-021-96634-8

**Published:** 2021-08-24

**Authors:** Kartick Sutradhar, Hari Om

**Affiliations:** Department of Computer Science and Engineering, Indian Institute of Technology (ISM) Dhanbad, Dhanbad, 826004 India

**Keywords:** Mathematics and computing, Computer science

## Abstract

The quantum secret sharing is an essential and fundamental technique for sharing a secret with the all participants in quantum cryptography. It can be used to design many complex protocols such as secure multiparty summation, multiplication, sorting, voting, etc. Recently, Song et al. have discussed a quantum protocol for secret sharing, which has (*t*, *n*) threshold approach and modulo *d*, where *t* and *n* denote the threshold number of participants and total number of participants, respectively. Kao et al. point out that the secret in the Song et al.’s protocol cannot be reconstructed without other participants’ information. In this paper, we discuss a protocol that overcomes this problem.

## Introduction

The quantum secret sharing includes a dealer and a group of *n* participants^1–7^. The dealer distributes the shares of a secret among *n* participants. When the dealer requires to retrieve the original secret, the *t* (threshold) number of participants will work together to retrieve it^8–11^. The quantum secret sharing can be used in various applications^12–20^, namely, secure multiparty summation^21,22^, multiplication^23^, comparison, sorting, voting, etc., as it preserves the secret from getting lost, damaged, or changed^24–26^. There have been discussed numerous protocols for sharing a secret in literature^8,27–31^. There are two approaches followed in quantum secret sharing protocols, namely, (*t*, *n*) and (*n*, *n*) threshold approaches. The first (*n*, *n*) threshold based quantum secret sharing protocol^31^ was discussed by Hillery et al. in 1999. Xiao et al.^32^ generalized this two-party protocol to a multi-party protocol. In 2005, the direct sharing of secret was discussed by Zhang^33^ based on quantum secure direct communication^34–36^. Qin et al. discussed a quantum secret sharing protocol^27^ based on (*n*, *n*) threshold in 2018. The first (*t*, *n*) threshold quantum based secret sharing protocol^28^ was introduced by Li et al. with modulo 2 in 2009. Ye et al.^37^ discussed the *d*-level quantum Fourier transform for secure quantum protocol in 2011. Yang et al. discussed a *d*-level and (*t*, *n*) threshold quantum based secret sharing protocol^29^ in 2013, that uses the quantum Fourier transform (*QFT*). Qin et al. introduced a (*t*, *n*) threshold quantum based secret sharing protocol^38^ with level-2 in 2015, using the operation of phase shift and creation of quantum entanglement^39,40^.

An (*t*, *n*) threshold quantum based secret sharing protocol with level-*d* was discussed by Song et al. in 2017 that used the *CNOT* operation, *QFT*, generalized Pauli operator, and inverse quantum Fourier transform (*IQFT*)^9^. This protocol includes a dealer and a group of participants. The dealer chooses one participant as a trusted reconstructor and SHA-1^41^ as the hash algorithm to evaluate the secret hash value. The dealer sends the secret’s hash value to a trusted reconstructor, who can recover the secret using a collision attack. Further, the trusted reconstructor cannot reconstruct the original secret from the *IQFT* operation^42^. The *IQFT* operation cannot sum up all the states. To recover the original secret, the trusted reconstructor needs other participants’ secret information. In 2020, Mashhadi improved the Song et al.’s protocol^43^ by using the *d*-level SUM operation, *QFT*, and *IQFT*. This protocol is efficient but it has high computation and communication costs due to the transmission of $(t-1)$ decoy particle, more number of *IQFT* operation, and SUM operation. Moreover, if the reconstructor is corrupted or dishonest, then the threshold number of participants cannot recover the secret in both the Mashhadi’s and Song et al.’s protocols. Hence, in these protocols, the reconstructor must be honest. In addition, similar to the Song et al.’s protocol, the trusted reconstructor may also recover the secret by performing the collision attack because the dealer sends the secret’s hash value to the trusted reconstructor directly. In this paper, we propose a new *d*-level quantum based secret sharing protocol using the (*t*, *n*) threshold approach that overcomes the above mentioned problems. We may summarize our contributions as follows.

- The reconstructor $Bob_1$ can reconstruct the original secret efficiently.
- The reconstructor $Bob_1$ cannot reveal the secret by performing the collision attack.
- The proposed protocol can also resist the coherent and collective attacks.
- The proposed protocol can also detect the eavesdropping by comparing the hash values of the secret even if the reconstructor transmits a fake secret to other participants after recovering the original secret.

## Preliminaries

Here, we introduce the Shamir’s secret sharing, *QFT*, and *IQFT*, which will be used in our proposed protocol.

### Shamir’s secret sharing^44^

This protocol has two phases as discussed below.

#### Sharing of secret

The dealer creates *n* shares of the secret using a polynomial *f*(*x*) of degree ($t-1$) and distributes *n* shares among *n* participants.

#### Reconstruction of secret

The threshold number of participants reconstructs the secret as follows.

1 $$f(x) = \sum _{v=1}^{t} f(x_v) \prod _{1 \le j \le t, j \ne v} \frac{x_j}{x_j - x_v} $$

### Quantum Fourier transform (*QFT*)^9^

The quantum Fourier transform (QFT) is defined as

$$\begin{aligned} QFT: |\alpha \rangle \rightarrow \frac{1}{\sqrt{d}} \sum _{\beta =0}^{d-1} e^{2\pi i\frac{\alpha }{d}\beta } |\beta \rangle . \end{aligned}$$

### Inverse quantum Fourier transform (*IQFT*)^9^

The inverse quantum Fourier transform (*IQFT*) is defined as

$$\begin{aligned} IQFT: |\beta \rangle \rightarrow \frac{1}{\sqrt{d}} \sum _{\alpha =0}^{d-1} e^{-2\pi i\frac{\beta }{d}\alpha } |\alpha \rangle . \end{aligned}$$

## Review of Song et al.’s protocol

Here, we review the Song et al.’s protocol. In this protocol, the dealer shares a secret *S* among *n* participants ${\mathcal {B}} = \{Bob_1, Bob_2, \dots , Bob_n \}$. From *n* participants, any one is selected by the dealer as a trusted reconstructor. We may consider here $Bob_1$ as a trusted reconstructor.

### Distribution of shares

The dealer selects an arbitrary polynomial *p*(*x*) of degree ($t-1$) such that $p(x) \in {\mathbb {Z}}_d$, where ${\mathbb {Z}}_d$ is a finite field. The ($t-1$)-degree polynomial may be defined as

$$\begin{aligned} p(x) = S + a_1x + \dots +a_{t-1}x^{t-1}. \end{aligned}$$

A non-zero value $x_i \in {\mathbb {Z}}_d$ is also selected by the dealer to compute *n* shares $p(x_i)$. The dealer encodes $p(x_i)'s$ using BB84 and sends the qubit string of $p(x_i)$ through a secure quantum channel to every participant $Bob_i, i=1,2,\ldots ,n$. The dealer chooses a hash algorithm *H*() to determine the hash value *H*(*S*) of the secret *S* and sends this hash value *H*(*S*) to the participant $Bob_1$.

### Reconstruction of secret

The secret is reconstructed by a certain number of participants using the following steps.

*Step 1* Participant $Bob_1$ (reconstructor) prepares a *t*-qudit particle $|l\rangle _1,|l\rangle _2, \dots , |l\rangle _t$, which contains *m* qubits, where $m = \lceil \log _{2}^{d} \rceil$. The participant $Bob_1$ applies the *QFT* on the particle $|l\rangle _1$ that results in the output state $|\varphi _1\rangle$, as follows.

2 $$\begin{aligned} \begin{aligned} |\varphi _1\rangle&= \left( QFT|l\rangle _1 \right) |l\rangle _2, |l\rangle _3, \dots , |l\rangle _t\\&= \left( \frac{1}{\sqrt{d}} \sum _{u=0}^{d-1} \omega ^{0.u} |u\rangle _1 \right) |l\rangle _2, |l\rangle _3, \dots , |l\rangle _t\\&= \left( \frac{1}{\sqrt{d}} \sum _{u=0}^{d-1} |u\rangle _1 \right) |l\rangle _2, |l\rangle _3, \dots , |l\rangle _t\\ \end{aligned} \end{aligned}$$

*Step 2* Participant $Bob_1$ again prepares a *v*-qudit particle $|l\rangle _v$, where $v=2, 3, \dots , t$, which contains *m* qubits, where $m = \lceil \log _{2}^{d} \rceil$. The participant $Bob_1$ applies the *d*-level *CNOT* gate^45^ on the particle $|l\rangle _v$, where $v= 2, 3, \dots , t$. After performing $(t-1)$ number of *CNOT* gates, the state $|\varphi _1\rangle$ becomes an entangled state $|\varphi _2\rangle$^39,40^ as follows.

3 $$\begin{aligned} \begin{aligned} |\varphi _2\rangle&= (CNOT((QFT|l\rangle _1),|l\rangle _2)) \otimes , \dots , \otimes (CNOT((QFT|l\rangle _1),|l\rangle _t))\\&= \frac{1}{\sqrt{d}} \sum _{u=0}^{d-1} |u\rangle _1 |u\rangle _2 |u\rangle _3, \dots , |u\rangle _t \end{aligned} \end{aligned}$$

*Step 3* Participant $Bob_1$ sends the particle $|u\rangle _v$ through a secure quantum channel to respective participant $Bob_v$, $v= 2, 3, \dots , t$.

*Step 4* Each participant $Bob_v$ evaluates the share’s shadow $(s_v)$, $v= 1, 2, \dots , t$, as follows.

4 $$\begin{aligned} s_v = f(x_v) \prod _{1\le j\le t, j\ne v} \frac{x_j}{x_j - x_v} \mod d \end{aligned}$$

*Step 5* The Pauli operator $(U_{0,s_v})$ is applied by each participant $Bob_v$ on their respective private particles $|u\rangle _v$, $v= 1, 2, \dots , t$, as follows.

5 $$\begin{aligned} U_{0,s_v} = \sum _{u=0}^{d-1} \omega ^{s_v.u} |u\rangle _{v\,\,v}\langle u| \end{aligned}$$

After performing the Pauli operator on each participant particle, the state $|\varphi _2\rangle$ extends as follows:

6 $$\begin{aligned} \begin{aligned} |\varphi _3\rangle&= \frac{1}{\sqrt{d}} \sum _{u=0}^{d-1} \omega ^{s_1.u} |u\rangle _1 \omega ^{s_2.u} |u\rangle _2 \omega ^{s_3.u} |u\rangle _3, \dots , \omega ^{s_t.u} |u\rangle _t \\&= \frac{1}{\sqrt{d}} \sum _{u=0}^{d-1} \omega ^{\left( \sum _{v=1}^{t} s_v\right) .u} |u\rangle _1 |u\rangle _2 |u\rangle _3, \dots , |u\rangle _t \end{aligned} \end{aligned}$$

*Step 6* Finally, the participant $Bob_1$ applies the *IQFT* on his private particle $|u\rangle _1$ and, based on computational basis, measures it to acquire the secret $p(0)'= \sum _{v=1}^{t} s_v \mod d$.

## Comments on Song et al.’s protocol

Here, we show the incorrectness of the reconstruction phase of the Song et al.’s protocol. Kao et al. point out that, without other participants’ information, $Bob_1$ can never retrieve the secret. Song et al. mention that $QFT(\sum _{v=1}^{t} s_v)$ is the qubit of $Bob_1$ in $|\varphi _1\rangle$. The participant $Bob_1$ evaluates *IQFT* over its particle $QFT(\sum _{v=1}^{t} s_v)$ and measures it on a computational base, where $Bob_1$ retrieves the secret $S' = \sum _{v=1}^{t} s_v$. We have the following observation.

7 $$\begin{aligned} \begin{aligned} |\phi _1\rangle =&\frac{1}{\sqrt{d}} \sum _{u=0}^{d-1}\omega ^{\left( \sum _{v=1}^{t}s_v\right) .u}|u\rangle _1 |u\rangle _2 \dots |u\rangle _t\\&\ne \frac{1}{\sqrt{d}}\left( \sum _{u=0}^{d-1}\omega ^{\left( \sum _{v=1}^{t}s_v\right) .u}|u\rangle _1\right) |u\rangle _2 \dots |u\rangle _t\\&= QFT\left( \sum _{v=1}^{t}s_v\right) |u\rangle _2 \dots |u\rangle _t. \end{aligned} \end{aligned}$$

The secret $S' = \sum _{v=1}^{t} s_v$ cannot be retrieved even when *IQFT* is performed over the particle $|l\rangle _1$ and measured computationally by $Bob_1$.

8 $$\begin{aligned} \begin{aligned} |\phi _2\rangle =&QFT \otimes I \otimes \dots \otimes I\left( \frac{1}{\sqrt{d}} \sum _{u=0}^{d-1}\omega ^{\left( \sum _{v=1}^{t}s_v\right) .u} |u\rangle _1 |u\rangle _2 \dots |u\rangle _t\right) \\&= \frac{1}{\sqrt{d}}\left( \sum _{u=0}^{d-1} IQFT\left( \omega ^{\left( \sum _{v=1}^{t}s_v\right) .u}|u\rangle _1\right) |u\rangle _2 \dots |u\rangle _t\right. \\&\left. \ne \frac{1}{\sqrt{d}} IQFT\left( \sum _{u=0}^{d-1}\omega ^{\left( \sum _{v=1}^{t}s_v\right) .u} |u\rangle _1\right) |u\rangle _2 \dots |u\rangle _t\right) \\&= |\sum _{v=1}^{t}s_v\rangle _1 |u\rangle _2 \dots |u\rangle _t. \end{aligned} \end{aligned}$$

For better understanding of the problem, consider an example, where $d = 3, t=2, n = 4$ and $S =2$. From step 5 of the reconstruction phase of the Song et al.’s protocol, we have

9 $$\begin{aligned} \begin{aligned} |\phi _3\rangle&=\frac{1}{\sqrt{3}}\sum _{u=0}^{2}\omega ^{2.u}|uu\rangle \\&\ne \frac{1}{\sqrt{3}}( \sum _{u=0}^{2}\omega ^{2.u} |u\rangle )|u\rangle \\&= QFT |2\rangle |u\rangle \end{aligned} \end{aligned}$$

On applying the inverse quantum Fourier transform *IQFT* over the particle $|u\rangle$, we get

10 $$\begin{aligned} \begin{aligned} |\phi _4\rangle&= QFT \otimes I \frac{1}{\sqrt{3}}(|00\rangle + \omega ^2|11\rangle + \omega |22\rangle )\\&=\frac{1}{\sqrt{3}}(QFT |0\rangle |0\rangle + \omega ^2 IQFT |1\rangle |1\rangle + \omega IQFT |2\rangle |2\rangle )\\&= \frac{1}{3}((|0\rangle + |1\rangle + |2\rangle )|0\rangle + \omega ^2(|0\rangle + \omega ^{-1}|1\rangle +\omega ^{-2}|2\rangle )|1\rangle + \omega (|0\rangle + \omega ^{-2}|1\rangle + \omega ^{-1}|2\rangle ) |2\rangle )\\&= \frac{1}{3}(|0\rangle (|0\rangle +\omega ^{2}|1\rangle + \omega |2\rangle ) + |1\rangle (|0\rangle + \omega |1\rangle +\omega ^{2}|2\rangle ) + |2\rangle (|0\rangle + |1\rangle + |2\rangle )). \end{aligned} \end{aligned}$$

The result to the equation comes out as $|0\rangle , |1\rangle$ or $|2\rangle$, not accurately $|2\rangle$.

### Attack on Song et al.’s protocol

The dealer chooses $Bob_1$ as a trusted reconstructor in the Song et al. protocol, and the hash algorithm $SHA-1$ to evaluate the secret’s hash value. After computing the hash value, the dealer transfers this hash value through a secure quantum channel to $Bob_1$. From this hash value, $Bob_1$ can easily reveal the secret by performing the collision attack.

## Proposed quantum secret sharing protocol

Here, we propose a new quantum secret sharing protocol that has (*t*, *n*) threshold and *d*-level. The distribution of the shares and the reconstruction of secret are its two main phases, as discussed below.

### Distribution of share

The dealer selects an arbitrary $(t-1)$-degree polynomial $p(x) \in {\mathbb {Z}}_d$, ${\mathbb {Z}}_d$ is a finite field, as follows:

$$\begin{aligned} p(x) = S + a_1x + \dots +a_{t-1}x^{t-1}. \end{aligned}$$

The dealer selects a non-zero value $x_i \in {\mathbb {Z}}_d$ to compute *n* shares $p(x_i)$, encodes $p(x_i)s$ using BB84 and sends the qubit string of $p(x_i)$ via a secure quantum channel to every participant $Bob_i, i=1,2,..,n$. Then, the dealer chooses a hash algorithm to determine the secret hash value ${\mathcal {H}}(S)$. After computing ${\mathcal {H}}(S)$, the dealer shares it using a polynomial $h(x)={\mathcal {H}}(S) + \gamma _1x + \gamma _2x^2 + \dots + \gamma _{t-1}x^{t-1}$ among *n* participants. Participant $Bob_i$ only learns the share $h(x_i)$, $i= 1, 2, \dots , n$.

### Reconstruction of the secret

Let ${\mathcal {B}}=\{Bob_1, Bob_2, \dots , Bob_t\}$ be a qualified subset of *t* participants. The dealer chooses a reconstructor participant from the qualified subset. In this phase, the dealer chooses $Bob_1$ as a reconstructor participant that recovers the secret and the secret hash value using the following steps:

*Step 1* Reconstructor $Bob_1$ prepares *t* qudit particle $|l\rangle _1,|l\rangle _2, \dots , |l\rangle _t$, which contains *m* qubits, $m = \lceil \log _{2}^{d} \rceil$. The participant $Bob_1$ applies the *QFT*^45^ on the particle $|l\rangle _1$. The output state $|\varphi _1\rangle$ is computed as follows.

11 $$\begin{aligned} \begin{aligned} |\varphi _1\rangle&= \left( QFT|l\rangle _1 \right) |l\rangle _2, |l\rangle _3, \dots , |l\rangle _t\\&= \left( \frac{1}{\sqrt{d}} \sum _{u=0}^{d-1} \omega ^{0.u} |u\rangle _1 \right) |l\rangle _2, |l\rangle _3, \dots , |l\rangle _t\\&= \left( \frac{1}{\sqrt{d}} \sum _{u=0}^{d-1} |u\rangle _1 \right) |l\rangle _2, |l\rangle _3, \dots , |l\rangle _t\\ \end{aligned} \end{aligned}$$

*Step 2* The participant $Bob_1$ prepares *v* qudit particle $|l\rangle _v$, $v=2, 3, \dots , t$ and this particle contains *m* qubits, $m = \lceil \log _{2}^{d} \rceil$. $Bob_1$ performs *d*-level *CNOT* gate on the particle $|l\rangle _v$, where $v= 2, 3, \dots , t$. After performing $(t-1)$ *CNOT* gates, the state $|\varphi _1\rangle$ becomes an entangled state $|\varphi _2\rangle$^39,40^ as follows.

12 $$\begin{aligned} \begin{aligned} |\varphi _2\rangle&= (CNOT((QFT|l\rangle _1),|l\rangle _2)) \otimes , \dots , \otimes (CNOT((QFT|l\rangle _1),|l\rangle _t))\\&= \frac{1}{\sqrt{d}} \sum _{u=0}^{d-1} |u\rangle _1 |u\rangle _2 |u\rangle _3, \dots , |u\rangle _t \end{aligned} \end{aligned}$$

*Step 3* $Bob_1$ sends the particle $|u\rangle _v$, $v= 2, 3, \dots , t$, to respective $Bob_v$ participants through a secure quantum channel.

*Step 4* Each participant $Bob_v$ evaluates the share’s shadow $(s_v)$, $v= 1, 2, \dots , t$.

13 $$\begin{aligned} s_v = f(x_v) \prod _{1\le j\le t, j\ne v} \frac{x_j}{x_j - x_v} \mod d \end{aligned}$$

*Step 5* The Pauli operator $(U_{0,s_v})$ applied by each participant $Bob_v$ on his private particle $|u\rangle _v$, $v= 1, 2, \dots , t$.

14 $$\begin{aligned} U_{0,s_v} = \sum _{u=0}^{d-1} \omega ^{s_v.u} |u\rangle _{v\,\,v}\langle u| \end{aligned}$$

After performing the Pauli operator on each participant particle, the state $|\varphi _2\rangle$ extends as follows:

15 $$\begin{aligned} \begin{aligned} |\varphi _3\rangle&= \frac{1}{\sqrt{d}} \sum _{u=0}^{d-1} \omega ^{s_1.u} |u\rangle _1 \omega ^{s_2.u} |u\rangle _2 \omega ^{s_3.u} |u\rangle _3, \dots , \omega ^{s_t.u} |u\rangle _t \\&= \frac{1}{\sqrt{d}} \sum _{u=0}^{d-1} \omega ^{\left( \sum _{v=1}^{t} s_v\right) .u} |u\rangle _1 |u\rangle _2 |u\rangle _3, \dots , |u\rangle _t \end{aligned} \end{aligned}$$

*Step 6* Each participant $Bob_v$ applies the *IQFT* on his private particle $|u\rangle _v$ and measures the result of *IQFT*. After measuring, each participant $Bob_v$ broadcasts the result of measurement.

*Step 7* Each participant $Bob_v$ computes the secret $p(0)'= \sum _{v=1}^{t} s_v \mod d$ by adding the measurement results.

*Step 8* Finally, all seven steps discussed above are again performed by the threshold number of participants *t* to reconstruct the secret hash value. The secret hash value $h(0)'=\sum _{r=1}^{t}\,g_r\,mod\,d$ is reconstructed by the participant $Bob_1$, where $g_r$ represents the hash value share’s shadow. The participant $Bob_1$ uses the hash algorithm $SHA-1$ to determine the hash value ${\mathcal {H}}(p(0)')$ and matches it with the secret’ hash value $h(0)'$. If $({\mathcal {H}}(p(0)')=h(0)')$, then the participant $Bob_1$ perceives that the threshold number of participants have executed the protocol honestly; otherwise, $Bob_1$ believes that the one or more corrupt participants have executed the protocol.

## Security analysis

In this section, we discuss the collision, coherent, and collective attacks, which can be resisted by the proposed protocol.

### Collision attack

An attacker uses the hash algorithm attack to generate the same secret hash value for two inputs in this attack. In the Song et al.’s^9^ and Mashhadi’s^43^ protocols, the $Bob_1$ can execute the collision attack to get the secret because the dealer sends the secret’s hash value to $Bob_1$ and hence it is not secure against the collision attack. Our protocol is secure against the collision attack because the dealer determines the secret hash value and shares this value among *n* participants. So, the reconstructor participant $Bob_1$ has no knowledge about the hash value and hence he is unable to execute the collision attack.

### Coherent attack

In this attack, an attacker creates an independent ancillary particle $|w\rangle$ and intercepts every participant’s particle $|l\rangle _v$ by jointly interacting with every qudit of participant $Bob_v, v=1,2,\dots , t$. On every participant’s particle $|l\rangle _v$, the attacker conducts the measurement process in computational basis. The attacker just gets *l* with $\frac{1}{d}$ probability from this calculation of particle $|l\rangle _v$. However, *l* does not hold any valuable data about the share’s shadow. Only the interacting particle $|l\rangle _v$ is known to the attacker in this case. As a result, the attacker cannot get the share’s shadow from the coherent attack.

### Collective attack

In a collective attack, an attacker communicates with each qudit by creating an individual ancillary particle and performing a measure all of the ancillary qudits at the same time to obtain the share’s shadow. Every qudit of participant $Bob_v, v=1,2,\dots , t$ is interacted with by an individual ancillary particle $|w\rangle$ created by the attacker. After communicating, the attacker obtains the particle $|l\rangle _v$ and conducts a joint calculation procedure in the computational basis to reveal the share’s shadow. Since the particle $|l\rangle _v$ does not hold any valuable data about the share’s shadow, the attacker cannot obtain any information about it from this joint calculation.

## Performance analysis

Here, we analyze the performance of the proposed protocol and compare with that of the Song et al.’s^9^, and Mashhadi’s^43^ protocols in terms of the security and cost. The Song em et al.’s protocol^9^ requires one *QFT* operation, *t* unitary operations, two hash operations, one *IQFT* operation, one measure operations, and transmit $(t-1)$ message particles. This protocol is not efficient because the *IQFT* cannot recover the original secret. The Mashhadi’s protocol^43^ needs one *QFT* operation, *t* unitary operations, two hash operations, *t* number of *IQFT* operations, $(t-1)$ SUM operations, *t* measure operations, and transmit $(t-1)$ message particles with $(t-1)$ decoy particles. However, our protocol requires one *QFT* operation, *t* unitary operations, two hash operations, $(t-1)$ *IQFT* operation, $(t-1)$ measure operations, and transmit $(t-1)$ number of message particles. Moreover, the Mashhadi’s protocol uses the SUM operation, more number of *IQFT* operation, and transmission of $(t-1)$ decoy particles; whereas, our protocol uses *CNOT* gate, less number of *IQFT* operation, and no transmission of the decoy particles. Hence, it has high cost as compared to our protocol. In addition, the proposed protocol is more cost effective, efficient, and secure as compared to the Song et al.’s^9^, and Mashhadi’s^43^ protocols. Table 1 shows the comparison of these protocols.

**Table 1:** Comparison of security and cost.

Performance parameter	Song et al.^9^	Mashhadi^43^	Proposed
SUM operation	–	$(t-1)$	–
Measure operation	1	*t*	$(t-1)$
Unitary operation	*t*	*t*	*t*
Decoy particle	–	$(t-1)$	–
Message particle	$(t-1)$	$(t-1)$	$(t-1)$
QFT operation	1	1	1
IQFT operation	1	*t*	$(t-1)$
Prevention of collision attack	No	No	Yes
Prevention of coherent attack	No	No	Yes
Prevention of collective attack	No	No	Yes

## Conclusion

In this paper, we have discussed a new (*t*, *n*) threshold protocol for quantum secret sharing in which the reconstructor can reconstruct the original secret efficiently. This protocol can execute the threshold number of participants without any trusted reconstructor participant. Further, the secret hash value and the secret are unknown to the reconstructor participant and he cannot execute the collision attack, but can correctly execute the proposed protocol. The proposed protocol can also resist the coherent and collective attacks.
